# Just the Facts

**DOI:** 10.1038/s42003-018-0030-x

**Published:** 2018-03-23

## Abstract

Scientific investigation is grounded in the objective pursuit of facts as guided by the scientific method. The process by which new results are vetted and communicated publicly—peer review—should be guided by similar principles.

A recent discussion among biologists on Twitter centered on open peer review, defined in this context as the disclosure of the referees’ identities to the authors (and usually to the readers, in the form of a published, signed review). One of the many facets of this issue that was discussed was the possibility of author retaliation against a named referee who provided a negative or critical review. This possibility is not hypothetical; participants in the discussion contributed their own real-life horror stories of just this kind of retaliation.

Whether in a review, rebuttal or public discussion of another scientist’s work, a guiding principle is the Golden Rule: do unto others as you would have them do unto you.

*Communications Biology* does not take a specific stance with regards to signed reviews. While we do not at present offer the option of transparent review (publication of referee reports with or without identification), we believe it is the referee’s choice whether to sign their reviews and thus be identified to both the authors and other referees. However, we do not think that the specific peer review model (open or anonymous) is intrinsically linked to the problem of retaliation against negative reviews. The same goes for the related issue of overly critical, or even hurtful, reviews.

The pursuit of knowledge is a messy business. Hypotheses turn out to be wrong, mistakes lead to serendipitous insights and sometimes apparent breakthroughs are later shown to be red herrings. This is all a natural part of the scientific endeavor. Science is also practiced by human beings. Unconscious biases can color even the most objective scientist’s views of their data. This is why all scientists work within a universally-recognized framework—the scientific method—to mitigate the effects of bias on the interpretation of experimental data. But what is the analogous framework for the interpersonal relationships between scientists in the practice and dissemination of science?

The problems are not simple, and therefore the solutions are not likely to be simple. Peer review is absolutely fundamental to the practice of science, and yet the incentives for providing thorough, useful and constructive reviews are not well-defined. Journals have a role to play in ensuring fair peer review, but institutions, funding bodies and hiring and tenure committees also have a role to play in ensuring that peer review is properly incentivized. There are no obvious quick fixes.

We do however believe there are a few ground rules that can be set to ensure the process is as fair as possible. Whether in a review, rebuttal or public discussion of another scientist’s work, a guiding principle is the Golden Rule: do unto others as you would have them do unto you. We believe peer review is most effective when it is focused on the facts and avoids personal attacks or inferences of motivation. The Golden Rule also prescribes respect, including respect for differences in culture and language. Science is international and a large proportion of scientists have the extremely challenging task of writing research articles in a second language. We urge caution when commenting on grammatical issues, and ask that referees avoid making negative inferences about the quality of the science based on the quality of the English. We also take a dim view of personal attacks in reviewer comments. Our editors reserve the right to return a review to the referee for modification before sending on to the authors. It should also go without saying that any speech that could be deemed derogatory or targeting a specific group, such as a gender or ethnicity, will not be tolerated.

Another guiding principle comes from the scientific method itself: one should always aim to disprove one’s own hypothesis. If a reviewer points out potential flaws or weaknesses in the data, crying foul as an author does nothing to further the pursuit of knowledge. As editors, we welcome appeals and discussion of reviewer comments, but our currency is data. Have you taken the referee’s comments to heart, tested their assertions scientifically, and still found that your hypothesis stands? If the referee’s comments are factually flawed, can you provide evidence (e.g., citations) or data to support your argument?

Civility and respect for anonymity (when it is desired) are the cornerstones of good peer review. As stated on the Nature Research peer review policy page, “We deplore any attempt by authors to confront reviewers or determine their identities.” Reviewers do not make decisions with regards to publication of manuscripts; editors do. If you feel that a referee has been unfair in his or her assessment of your work, the correct course of action is always to contact the editor.

At *Communications Biology*, we have been overwhelmed by the positive support of the biological community. In virtually every case, constructive reviews have led to improvements the manuscripts we handle. But to be clear: any instances of author or referee misconduct in the peer review process that are brought to our attention will be treated seriously and in accordance with the wider Nature Research policy on publication ethics. We reserve the right to contact funders, regulatory bodies and the author’s or referee’s institutions in cases of suspected research or publishing misconduct, which includes intimidation of referees. We hope the need to do so will never arise.

Finally, it’s important to keep in mind that although there are many examples of bad behavior in peer review, our experience as editors has shown that the majority of the time, peer review is a productive and positive process. Peer referees apply their expertise, from a necessary distance, in order to help the authors—their peers and colleagues—identify their own blind spots prior to publication. Bias in science will be inevitable as long as scientists remain human, but when carried out with the goal of improving the publication through objectivity and honesty, peer review helps to minimize that bias and bring the facts to light.

